# Characteristics of Cell Damage at the Division Stage and Nuclear Aberrations in the Buccal Epithelium Cytogram of Individuals with Impaired Carbohydrate Metabolism

**DOI:** 10.3390/diseases14100381

**Published:** 2026-10-16

**Authors:** Irina Usmanova, Irina Lakman, Svetlana Kolbetsova, Yulianna Enina, Olesya Kytko, Elizaveta Kytko, Yuriy Vasil’ev

**Affiliations:** 1 Department of Therapeutic Dentistry, Bashkir State Medical University of the Russian Ministry of Health, Ufa 450008, Russia; irinausma@mail.ru; 2 Ufa University of Science and Technology, Ufa 450076, Russia; lackmania@mail.ru; 3 Sakhautdinov City Clinical Hospital No. 21, Ufa 450071, Russia; svetlana.k.vr@yandex.ru; 4 Sechenov First Moscow State Medical University of the Ministry of Health of the Russian Federation (Sechenov University), Moscow 119048, Russia; enina_yu_i@staff.sechenov.ru (Y.E.); kytko_o_v@staff.sechenov.ru (O.K.); kytko_e_n@student.sechenov.ru (E.K.)

**Keywords:** buccal epithelium, cytogram, nuclear aberrations, diabetes mellitus, carbohydrate metabolism, metabolic syndrome

## Abstract

Aim: To compare the frequency of cell damage at the division stage, apoptosis, and necrosis/nuclear destruction in the buccal epithelial cytogram of individuals with impaired carbohydrate metabolism. Materials and Methods: In this observational cross-sectional study, buccal epithelial cytograms were obtained from 52 patients with type 2 diabetes mellitus (T2DM), 26 patients with metabolic syndrome (MS) and impaired carbohydrate metabolism (prediabetes), and 27 patients with MS without impaired carbohydrate metabolism. The smears were stained with eosin-methylene blue followed by Romanowsky staining (azure-eosin solution). Venous blood samples were tested for fasting glucose, 2 h post-load glucose, and glycated hemoglobin (HbA1c, %). The frequency of cell damage per 1000 cells in the cytogram was expressed as the median and interquartile range. Between-group comparisons were performed with the non-parametric Kruskal–Wallis test; when differences were present, Dunn’s post hoc pairwise tests were applied. Spearman’s rank correlation coefficient was calculated to assess the relationship between the HbA1c level and the frequency of nuclear destruction parameters in buccal epithelial cells per 1000 cells. Results: The median frequency of nuclear vacuolization in the buccal epithelial cytogram of T2DM patients was 1.5-fold and 2.5-fold higher than in MS patients with prediabetes (*p* < 0.001) and in MS patients without impaired carbohydrate metabolism (*p* < 0.001), respectively. Binucleated cells were more frequent in the cytograms of MS patients with prediabetes than in those of MS patients without impaired carbohydrate metabolism (*p* = 0.008). Nuclear protrusions were observed more often in the buccal epithelium of T2DM patients than in that of MS patients without impaired carbohydrate metabolism (*p* = 0.015). Karyopyknosis was less frequent in T2DM patients than in MS patients without impaired carbohydrate metabolism (*p* = 0.006) and did not differ significantly from the values in MS patients with prediabetes (*p* = 0.363). Karyorrhexis in the buccal epithelial cytogram was 34-fold higher in T2DM patients (*p* < 0.001) and 6-fold higher in MS patients with prediabetes (*p* < 0.01) than in MS patients without impaired carbohydrate metabolism. Karyolysis was significantly more frequent in T2DM patients than in MS patients without impaired carbohydrate metabolism (*p* = 0.014) and did not differ significantly from the values in MS patients with prediabetes (*p* > 0.1). Conclusions: In patients with MS, T2DM and prediabetes were associated with a higher frequency of nuclear abnormalities and degenerative changes in buccal epithelial cells. Because oral hygiene and periodontal inflammation indices were also worse in these groups and no multivariable adjustment was performed, these associations cannot be attributed independently to hyperglycemia and should be regarded as hypothesis-generating. Buccal epithelial cytology may be considered a simple candidate, non-invasive complementary indicator of impaired carbohydrate metabolism that requires further validation, rather than a standalone diagnostic or screening tool; this preliminary observational study lacked a healthy (non-MS) control group and diagnostic accuracy analyses (ROC curves, sensitivity/specificity, cut-off values), which are needed before any clinical application.

## 1. Introduction

Impaired carbohydrate metabolism is a common metabolic condition characterized by hyperglycemia, which leads to secondary disturbances of lipid and protein metabolism and to cellular changes. As early as 2008, Jajarm H.H. et al. demonstrated significant differences in the cytograms of oral epithelial cells in patients with type 2 diabetes mellitus (T2DM) compared to healthy individuals [1]. More recent studies presented data confirming significant differences in the frequency of apoptosis and necrosis of buccal epithelial cells, particularly karyorrhexis and nuclear vacuolization, between healthy individuals and individuals with diabetes mellitus, regardless of its type [2]. Studies have also confirmed the presence of significantly more frequent abnormalities in the buccal epithelial cytogram, such as karyorrhexis, karyolysis, and nuclear apoptosis, in individuals with diabetes mellitus [3]. However, there are virtually no studies comparing buccal epithelial cytograms in individuals with prediabetes and metabolic syndrome. A comparative analysis of buccal epithelial cytograms published by Russian researchers in obese adolescents (with a BMI greater than 2 standard deviations above the mean for this age group) with those in normal-weight adolescents showed differences in the frequency of binucleated cells and the apoptotic index [4]. Intracellular changes in buccal epithelial cells have been proposed as an early indicator of oral mucosal and periodontal pathology in the context of somatic diseases [5].

Recent evidence further clarified the cellular mechanisms underlying this damage: hyperglycemia-associated oxidative stress and impaired DNA-repair proficiency were demonstrated in the peripheral lymphocytes and buccal mucosal cells of patients with type 1 diabetes, supporting a genotoxic basis for the cytological abnormalities observed in diabetic oral epithelium [6]. Beyond diabetes, exfoliative cytology of the oral mucosa is increasingly recognized as a versatile, minimally invasive platform for detecting a broad range of systemic diseases, not only oral and systemic malignancies but also metabolic and inflammatory conditions, reflecting growing interest in its use as a monitoring tool outside oncology [7]. In parallel, oral fluid and mucosal biomarker discovery efforts—including salivary and cytological markers linked to glycemic control and chronic low-grade inflammation—are expanding the panel of non-invasive indicators available for the early detection and monitoring of impaired carbohydrate metabolism, although most of these markers still require validation against accepted diagnostic reference standards before clinical adoption [8].

Therefore, there is interest in identifying differences in buccal epithelial cytograms between individuals with metabolic syndrome (MS) with and without impaired carbohydrate metabolism (prediabetes/diabetes). Such an analysis could generate hypotheses for the development of early and accessible screening approaches for impaired carbohydrate metabolism in individuals with MS, which would then require validation in appropriately designed diagnostic accuracy studies; this determined the purpose and relevance of our study.

The aim of the study was to compare the frequency of cell damage at the division stage, apoptosis, and cell necrosis/nuclear destruction in the buccal epithelial cytogram of individuals with impaired carbohydrate metabolism.

## 2. Materials and Methods

**Figure 1:**
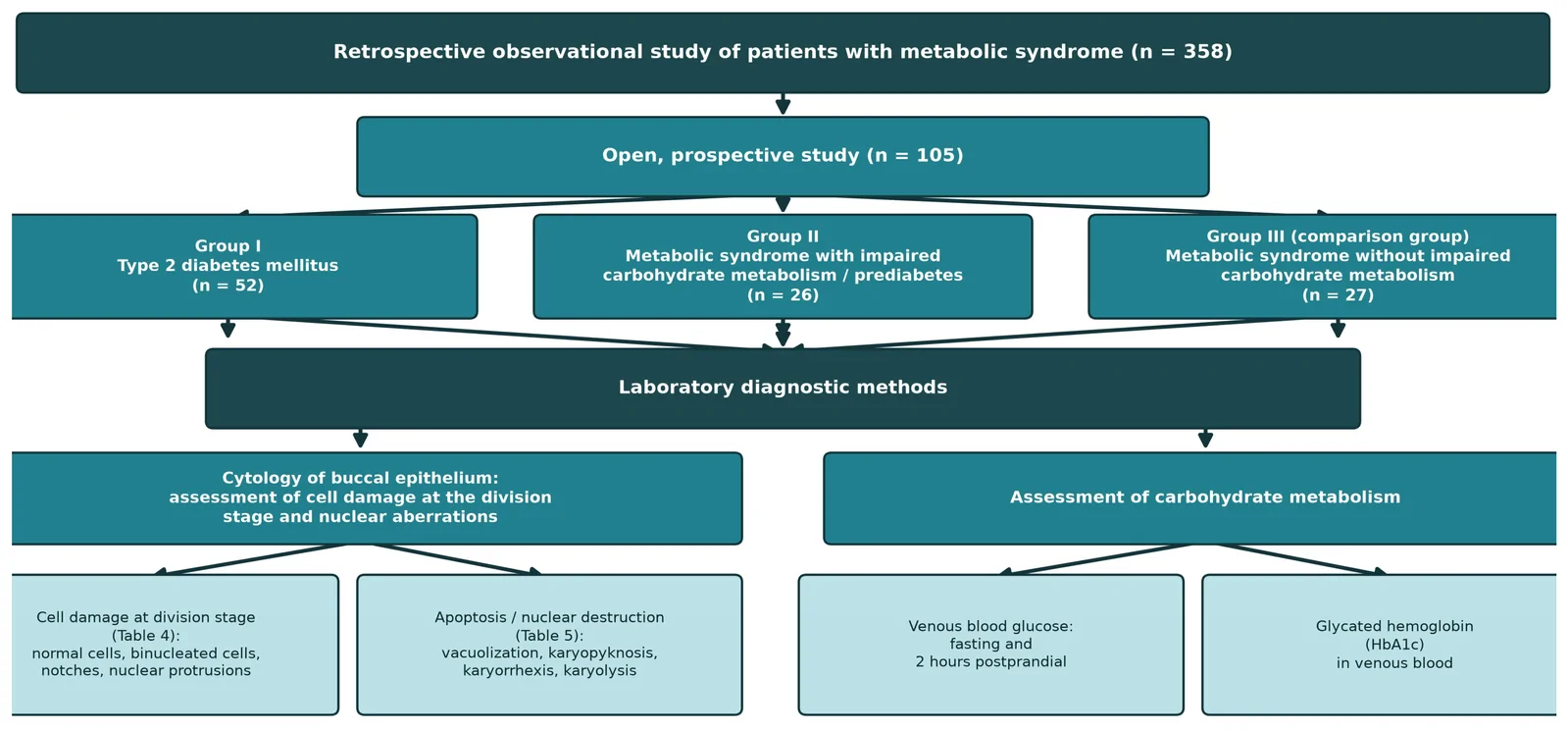
Research design.

The study had a two-stage design (Figure 1). At the first stage, the medical records of patients with metabolic syndrome were reviewed retrospectively, and the patients were stratified according to the presence and severity of impaired carbohydrate metabolism. At the second stage, consenting patients aged 19–62 years from each stratum were enrolled in an open, prospective, cross-sectional examination, during which buccal epithelial samples, unstimulated oral fluid, and venous blood were collected on a single occasion according to a standardized protocol. Thus, group allocation was based on retrospective stratification of medical records, whereas all cytological and laboratory data were collected prospectively. Three groups were formed: Group I—52 patients with T2DM; Group II—26 patients with MS and impaired carbohydrate metabolism (prediabetes); Group III (comparison group)—27 patients with MS without impaired carbohydrate metabolism.

### 2.1. Group Formation Criteria Were as Follows

Group III (comparison group), MS without impaired carbohydrate metabolism: presence of MS components (abdominal obesity, arterial hypertension, elevated triglycerides and low-density lipoprotein cholesterol, reduced high-density lipoprotein cholesterol); fasting venous plasma glucose < 6.1 mmol/L; 2 h post-load plasma glucose during the oral glucose tolerance test (OGTT) < 7.8 mmol/L; HbA1c < 6.0%; and no previous diagnosis of diabetes mellitus or use of glucose-lowering drugs.

Group II, MS with impaired carbohydrate metabolism (prediabetes): presence of MS components and at least one of the following—impaired fasting glucose (IFG), defined as fasting venous plasma glucose ≥ 6.1 and <7.0 mmol/L with 2 h post-load plasma glucose < 7.8 mmol/L; impaired glucose tolerance (IGT), defined as 2 h post-load plasma glucose ≥ 7.8 and ≤11.1 mmol/L with fasting plasma glucose < 7.0 mmol/L; a combination of IFG and IGT; or HbA1c ≥ 6.0% and <6.5%.

Group I, T2DM: presence of MS components and at least one of the following—fasting venous plasma glucose ≥ 7.0 mmol/L (on two occasions or in the presence of symptoms); 2 h post-load plasma glucose > 11.1 mmol/L; HbA1c ≥ 6.5%; or a previously confirmed diagnosis of T2DM and/or current use of glucose-lowering drugs.

The diagnosis of metabolic syndrome was established in accordance with the international criteria of the International Diabetes Federation (IDF, 2006; Alberti et al., 2006) [9]. A mandatory criterion was the presence of central (abdominal) obesity—defined by a waist circumference of ≥94 cm for men and ≥80 cm for women (or a BMI > 30 kg/m^2^)—combined with any two of the following four factors: elevated triglyceride levels (≥1.7 mmol/L (150 mg/dL) or specific lipid-lowering therapy); reduced HDL cholesterol levels (<1.03 mmol/L (40 mg/dL)) for men and <1.29 mmol/L (50 mg/dL) for women, or specific therapy); elevated blood pressure (systolic BP ≥ 130 mmHg or diastolic BP ≥ 85 mmHg, or use of antihypertensive medication); and elevated fasting plasma glucose (≥5.6 mmol/L (100 mg/dL) or previously diagnosed type 2 diabetes mellitus). Participants were recruited via consecutive (all-comer) selection as they presented for care, resulting in unequal group sizes. Eighteen individuals declined to participate in the study.

### 2.2. Exclusion Criterion: Absence of MS Components

The study protocol was approved by the Local Ethics Committee of Bashkir State Medical University (Protocol No. 5 dated 15 May 2024). The study was conducted at the clinic of Bashkir State Medical University and City Clinical Hospital No. 21, Ufa. Written informed consent was obtained from each participant prior to enrollment.

For all patients, clinical and demographic data were collected, and oral hygiene status (simplified oral hygiene index, OHI-S) and the clinical status of the periodontal tissues (papillary–marginal–alveolar index, PMA) were assessed. For each patient, a sample of unstimulated mixed oral fluid was collected for glucose measurement, buccal epithelium was sampled for cytological examination, and hygiene status was assessed at the same visit, in the morning between 8:00 and 8:30 local time.

Glucose levels were determined in oral fluid samples—collected over a 15 min period in the fasting state and without prior mouth rinsing—using the “Glucose GOD” kit (JSC Vector-Best, Novosibirsk, Russia), which has a measurement range of 0.06–30 mmol/L. To ensure that the measured glucose concentrations in the oral fluid samples fell within the linearity range of the kit (0.5–56 mmol/L), the volume of the sample used for analysis was increased. Glucose in the oral fluid samples was measured by a biochemical assay in a microplate format using “Glucose-Novo” reagents (Vector-Best).

Buccal epithelial samples were collected with sterile disposable cytobrushes from the buccal mucosa above the occlusion line.

Before sampling, the clinical condition of the mucosa was assessed for obvious inflammatory changes, fissures, ulcerations, and white plaque. Patients were instructed to refrain from eating and drinking (including tea, coffee, and juice), tooth brushing, and chewing gum for 60–90 min before sampling, and from smoking for 4 h before sampling. Immediately before sampling, the patient had to rinse the mouth thoroughly 2–3 times with clean water. The cytobrush was applied to the buccal mucosa above the occlusion line with 10–20 light strokes under slight pressure. The material obtained was transferred to sterile glass slides with the cytobrush, air-dried, and stained with eosin-methylene blue followed by Romanowsky staining (azure-eosin solution). The following cytological parameters of the buccal epithelial cells were then evaluated: the frequency of normal cells, cells with nuclear vacuolization, cells with condensed chromatin (karyopyknosis—a dense, homogeneous nucleus indicating apoptosis or cell damage), karyorrhexis (fragmentation of the nucleus), karyolysis, binucleated cells, cells with nuclear notches, and cells with nuclear protrusions (“broken egg” appearance—a micronucleus-like body connected to the main nucleus by a nucleoplasmic bridge). In accordance with international scoring guidelines for buccal micronucleus cytome assays (HUMNxl), nuclear protrusions/buds were distinguished from micronuclei: nuclear buds are nucleoplasmic evaginations that remain connected to the main nucleus by a visible nucleoplasmic bridge, whereas micronuclei are independent, extranuclear chromatin bodies with no connection to the main nucleus; the two categories were scored as distinct morphological entities and are not used interchangeably in this study. Standardization of the cytogram samples was carried out according to the approach described by Sánchez-Alarcón J. et al. (2026) [10].

The buccal epithelial preparations of each patient were evaluated in parallel by two cytologists from the same medical center, who were blinded to the group allocation of the patients. In cases of disagreement regarding nuclear abnormalities, the slides were reviewed jointly until a consensus was reached.

#### Criteria for the Identification of Nuclear Abnormalities Were as Follows

Karyorrhexis was identified by nuclear fragmentation; karyolysis by a nucleus that loses its ability to stain, swells, becomes pale, and finally disappears, leaving a shadow in the cytoplasm; binucleation by the presence of two complete nuclei or a double nucleus. According to the study protocol, 1000 cells were evaluated in each preparation, and the frequency of each type of abnormality was expressed per 1000 cells.

On the day of buccal epithelium sampling, fasting and 2 h post-load venous plasma glucose levels were measured, and HbA1c was determined by high-performance liquid chromatography. The clinical and laboratory study design is shown in Figure 1.

Statistical analysis was performed using R 4.4.3 (the ‘stats’ package, version 4.4.3). The frequency of cell damage per 1000 cells was expressed as the median and interquartile range. The Kruskal–Wallis test was used for between-group comparisons. If the null hypothesis was rejected at *p* ≤ 0.05, Dunn’s post hoc pairwise tests were applied, with the significance threshold set at *p* < 0.016 (0.05/3, Bonferroni correction for three pairwise comparisons). Spearman’s rank correlation coefficient was used to assess relationships between HbA1c levels and nuclear destruction parameters per 1000 cells. These analyses were considered the primary analyses of the study.

In addition, an exploratory, secondary compositional analysis was performed on a defined four-part sub-composition of cytological findings—normal cells, karyopyknosis, karyorrhexis, and karyolysis, expressed as counts per 1000 cells—considered jointly as relative proportions of one another rather than as an exhaustive partition of all 1000 assessed cells. To compare the multivariate profiles of these compositional data among the three groups, PERMANOVA was applied after centered log-ratio (CLR) transformation of the data and calculation of the Aitchison distance. PERMANOVA was chosen because counts of cells with abnormalities per 1000 cells are compositional in nature. The CLR transformation converts relative data (effectively proportions per 1000 cells) into logarithms of the ratios of individual indicators to the geometric mean of all assessed features for each sample. Based on the transformed data, the Aitchison distance was calculated, representing the Euclidean distance in log-ratio coordinates. Post hoc pairwise PERMANOVA tests with Bonferroni correction were also conducted to compare specific pairs of groups. The compositional analysis was performed in R using the “compositions” and “vegan” packages (version 2.7.6). Prior to the centered log-ratio (CLR) transformation, zero values within the four-component subcomposition (normal cells, karyopyknosis, karyorrhexis, and karyolysis) were imputed using a multiplicative simple replacement strategy via the “cmultRepl” function from the “zCompositions” package (version 1.6.2)in R. This approach avoids the mathematical indeterminacy of log-ratios associated with zero values while preserving the subcomposition’s covariance structure.

The study we conducted should be interpreted exclusively as exploratory and hypothesis-generating.

## 3. Results

The study included 105 patients with MS, divided into three groups: Group I (*n* = 52), T2DM; Group II (*n* = 26), MS with impaired carbohydrate metabolism (prediabetes); and Group III (comparison group, *n* = 27), MS without impaired carbohydrate metabolism. The sex distribution (female to male) in Groups I, II, and III was 25:27, 20:6, and 24:3, respectively. The median age was 36.5 (25.5–53), 38 (36–52), and 38.5 (35–50) years, respectively. The median fasting venous plasma glucose levels in Groups I, II, and III were 10.6 (7.0–14.2), 5.2 (4.6–5.5), and 4.65 (4.3–5.3) mmol/L, respectively. The median 2 h post-load glucose levels were 9.1 (8.0–9.8), 8.1 (7.9–8.7), and 6.2 (5.4–6.9) mmol/L, respectively, and the median HbA1c levels were 9.7 (8.8; 10.5), 6.3 (5.2; 7.3), and 5.5 (5.1; 6.1) %, respectively. A detailed comparison of the clinical and demographic characteristics of the groups is provided in Table 1. As expected from the group formation criteria, the groups differed significantly in fasting venous plasma glucose, 2 h post-load venous plasma glucose, and HbA1c (Kruskal–Wallis test; *p* < 0.001, *p* < 0.001 and *p* = 0.001, respectively). High-sensitivity C-reactive protein (hs-CRP) in venous plasma, analyzed as a marker of inflammation, also differed significantly across the groups (*p* < 0.001): the lowest median hs-CRP level was observed in Group III, the comparison group without impaired carbohydrate metabolism (3.8 mg/L), compared with 8.0 mg/L in Group I (T2DM) and 5.0 mg/L in Group II (prediabetes). The groups did not differ in smoking status (*p* > 0.2). The groups also differed significantly in sex distribution (χ^2^ = 15.065, *p* < 0.001), with the proportion of women lowest in Group I (T2DM, 48.1%) and highest in Group III (comparison group, 88.9%), and in age among men (*p* = 0.048), whereas age did not differ significantly among women (*p* = 0.061); these baseline imbalances were taken into account in the interpretation of the findings (see Section 6).

Group assignment (Group I, T2DM; Group II, prediabetes; Group III, comparison group) was based on meeting at least one of several alternative criteria (fasting venous plasma glucose, 2 h post-load glucose, HbA1c, or a previously confirmed diagnosis/treatment), as defined in Section 2 (Group formation criteria). Because patients could be assigned to a group on the basis of a glucose criterion alone, the group-level HbA1c distribution can include individual values outside the HbA1c range nominally associated with that group; this reflects the multi-criterion classification scheme rather than misclassification, but the resulting overlap in HbA1c values between Group II and Group I should be taken into account when interpreting Table 1.

Table 2 compares the oral hygiene (OHI-S) and periodontal (PMA) indices between the study groups. According to the Kruskal–Wallis test, both indices differed significantly between the groups (*p* < 0.01); the values were highest in the T2DM group and lowest in the comparison group, indicating poorer oral hygiene and more pronounced gingival inflammation in patients with impaired carbohydrate metabolism.

Table 3 presents the between-group comparison of glucose levels in unstimulated mixed oral fluid. Pairwise comparisons revealed significant differences in oral fluid glucose levels between the groups: the median was 800 μmol/L in the T2DM group, 300 μmol/L in the prediabetes group, and 150 μmol/L in patients with MS without impaired carbohydrate metabolism.

**Figure 2:**
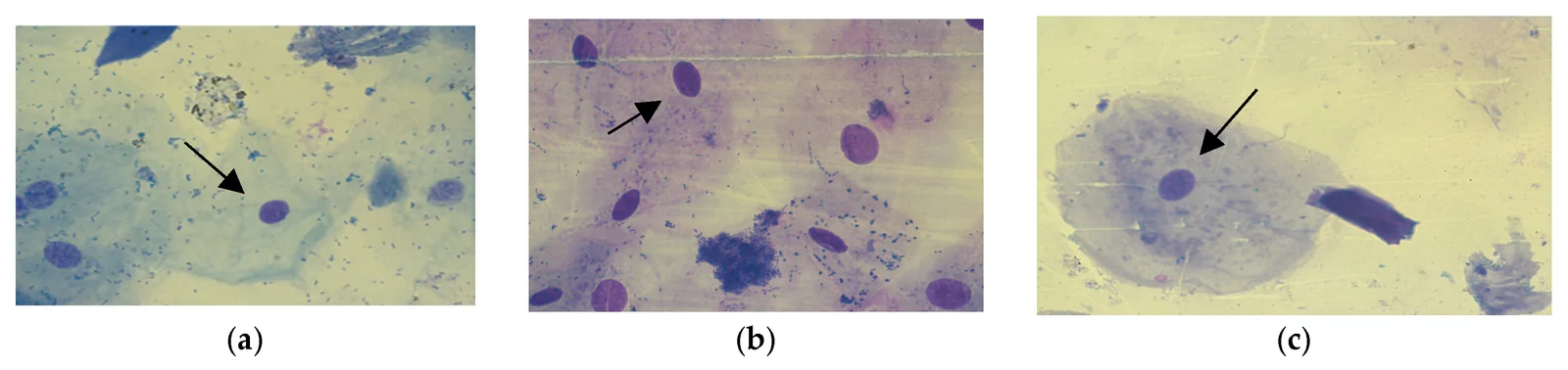
(**a**) Normal cell in the buccal epithelial cytogram, Group I (T2DM). May–Grünwald eosin-methylene blue staining with Romanowsky azure-eosin counterstain. Magnification ×1000. (**b**) Normal cell in the buccal epithelial cytogram, Group II (MS with impaired carbohydrate metabolism, prediabetes). May–Grünwald eosin-methylene blue staining with Romanowsky azure-eosin counterstain. Magnification ×1000. (**c**) Normal cell in the buccal epithelial cytogram, Group III (MS without impaired carbohydrate metabolism, comparison group). May–Grünwald eosin-methylene blue staining with Romanowsky azure-eosin counterstain. Magnification ×1000.

Analysis of the buccal epithelial cytograms revealed significant differences in the frequency of normal cells per 1000 cells among all three study groups (Table 4, Figure 2a–c), which were confirmed by Dunn’s post hoc tests adjusted for multiple comparisons (*p* < 0.001 for all pairwise comparisons). The median frequency of normal cells in the T2DM group was almost 10-fold lower than in patients with MS without impaired carbohydrate metabolism (comparison group), whereas in patients with MS and prediabetes it was approximately 2-fold lower (Table 4).

**Figure 3:**
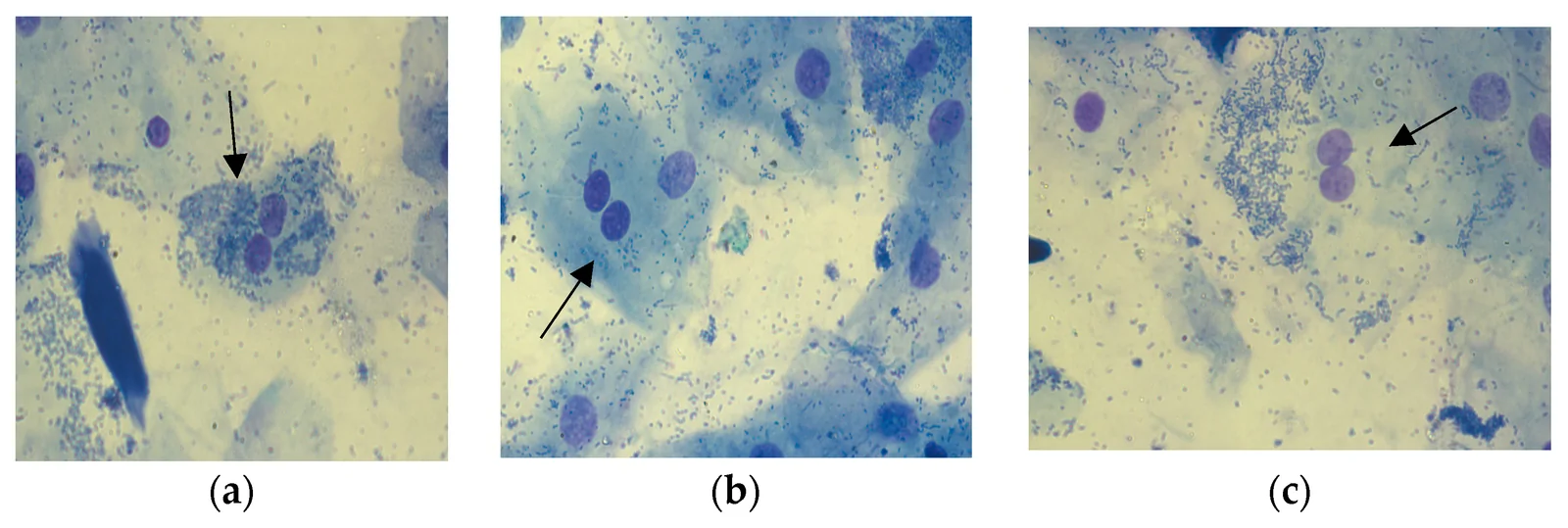
(**a**) Binucleated cell in the buccal epithelial cytogram, Group I (T2DM). May–Grünwald eosin-methylene blue staining with Romanowsky azure-eosin counterstain. Magnification ×1000. (**b**) Binucleated cell in the buccal epithelial cytogram, Group II (MS with impaired carbohydrate metabolism, prediabetes). May–Grünwald eosin-methylene blue staining with Romanowsky azure-eosin counterstain. Magnification ×1000. (**c**) Binucleated cell in the buccal epithelial cytogram, Group III (MS without impaired carbohydrate metabolism, comparison group). May–Grünwald eosin-methylene blue staining with Romanowsky azure-eosin counterstain. Magnification ×1000.

Binucleated cells were significantly more frequent in the buccal epithelium of patients with MS and prediabetes than in that of patients with MS without impaired carbohydrate metabolism (*p* = 0.008; Table 4, Figure 3a–c).

**Figure 4:**
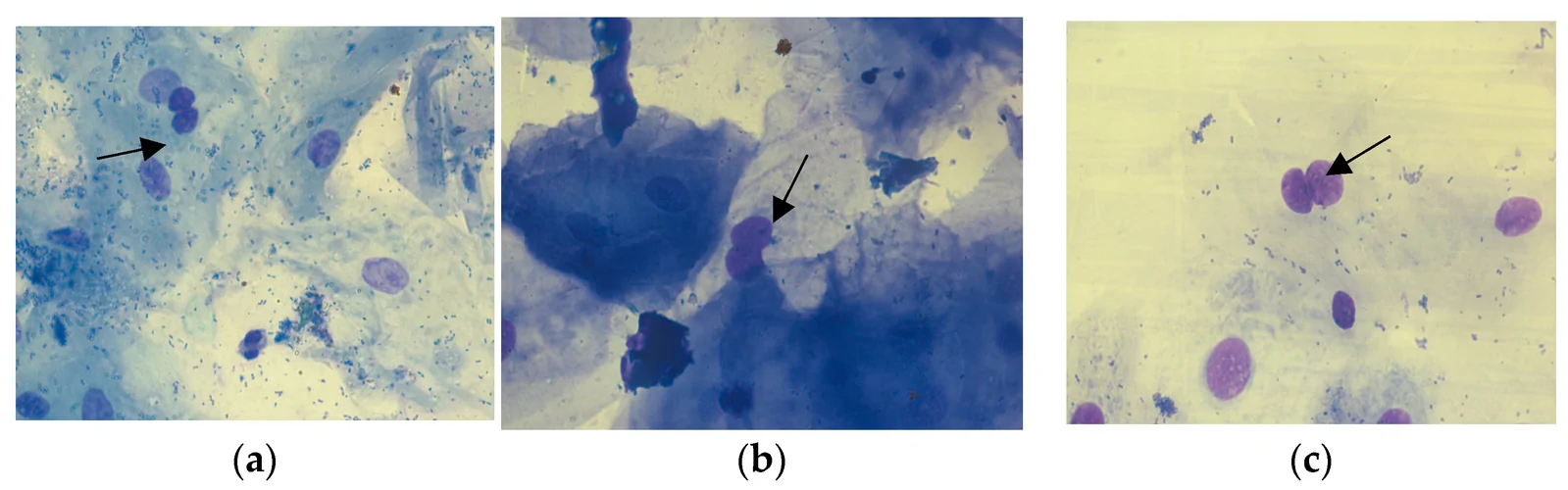
(**a**) Buccal epithelial cell with a notched nucleus, Group I (T2DM). May–Grünwald eosin-methylene blue staining with Romanowsky azure-eosin counterstain. Magnification ×1000. (**b**) Buccal epithelial cell with a notched nucleus, Group II (MS with impaired carbohydrate metabolism, prediabetes). May–Grünwald eosin-methylene blue staining with Romanowsky azure-eosin counterstain. Magnification ×1000. (**c**) Buccal epithelial cell with a notched nucleus, Group III (MS without impaired carbohydrate metabolism, comparison group). May–Grünwald eosin-methylene blue staining with Romanowsky azure-eosin counterstain. Magnification ×1000.

Nuclear notches (notched nuclei) were almost twice as frequent in the buccal epithelial cytograms of patients with T2DM and of patients with MS and prediabetes as in those of patients with MS without impaired carbohydrate metabolism. The Kruskal–Wallis test indicated a significant between-group difference (*p* = 0.024), but Dunn’s post hoc tests adjusted for multiplicity revealed no significant pairwise differences (p_1,2_ = 0.612; p_2,3_ = 0.048; p_1,3_ = 0.025; none of these values reached the Bonferroni-adjusted significance threshold of *p* < 0.016) (Table 4, Figure 4a–c).

**Figure 5:**
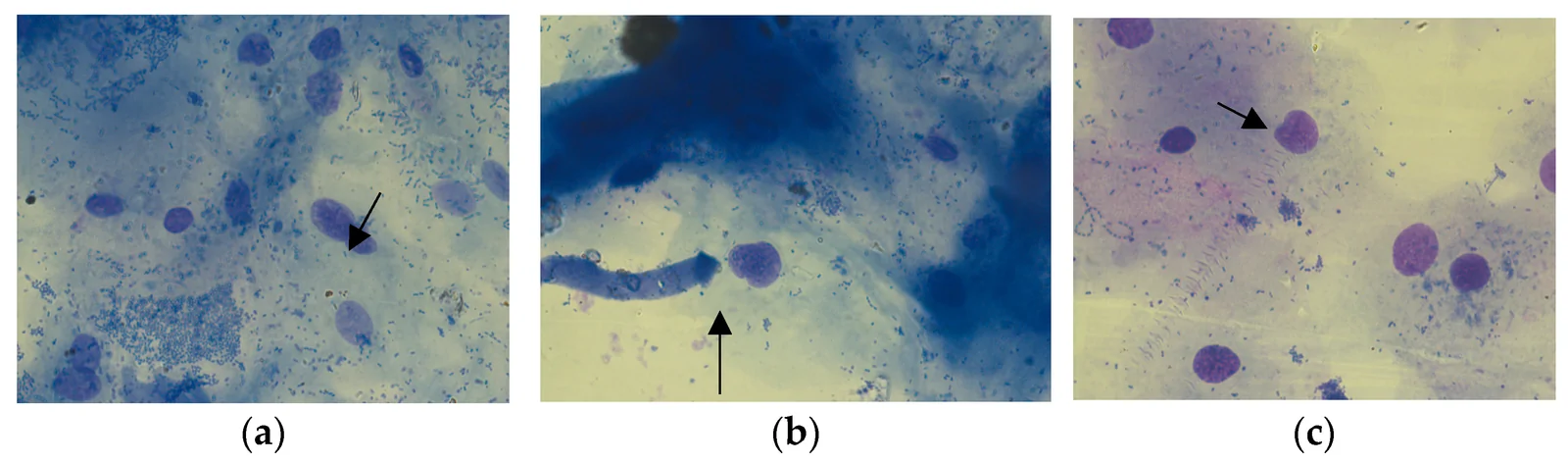
(**a**) Nuclear protrusion in the buccal epithelial cytogram, Group I (T2DM). May–Grünwald eosin-methylene blue staining with Romanowsky azure-eosin counterstain. Magnification ×1000. (**b**) Nuclear protrusion in the buccal epithelial cytogram, Group II (MS with impaired carbohydrate metabolism, prediabetes). May–Grünwald eosin-methylene blue staining with Romanowsky azure-eosin counterstain. Magnification ×1000. (**c**) Nuclear protrusion in the buccal epithelial cytogram, Group III (MS without impaired carbohydrate metabolism, comparison group). May–Grünwald eosin-methylene blue staining with Romanowsky azure-eosin counterstain. Magnification ×1000.

Nuclear protrusions (“nuclear buds”) were significantly more frequent in the buccal epithelial cytograms of patients with T2DM than in those of patients with MS without impaired carbohydrate metabolism (*p* = 0.015; Table 4, Figure 5a–c and Figure 14).

Table 5 and Figure 6, Figure 7, Figure 8, Figure 9, Figure 10, Figure 11, Figure 12, Figure 13 and Figure 14 present the frequency of apoptosis and cell necrosis/nuclear destruction in the buccal epithelial cytogram per 1000 cells in the main clinical groups and the comparison group.

**Figure 6:**
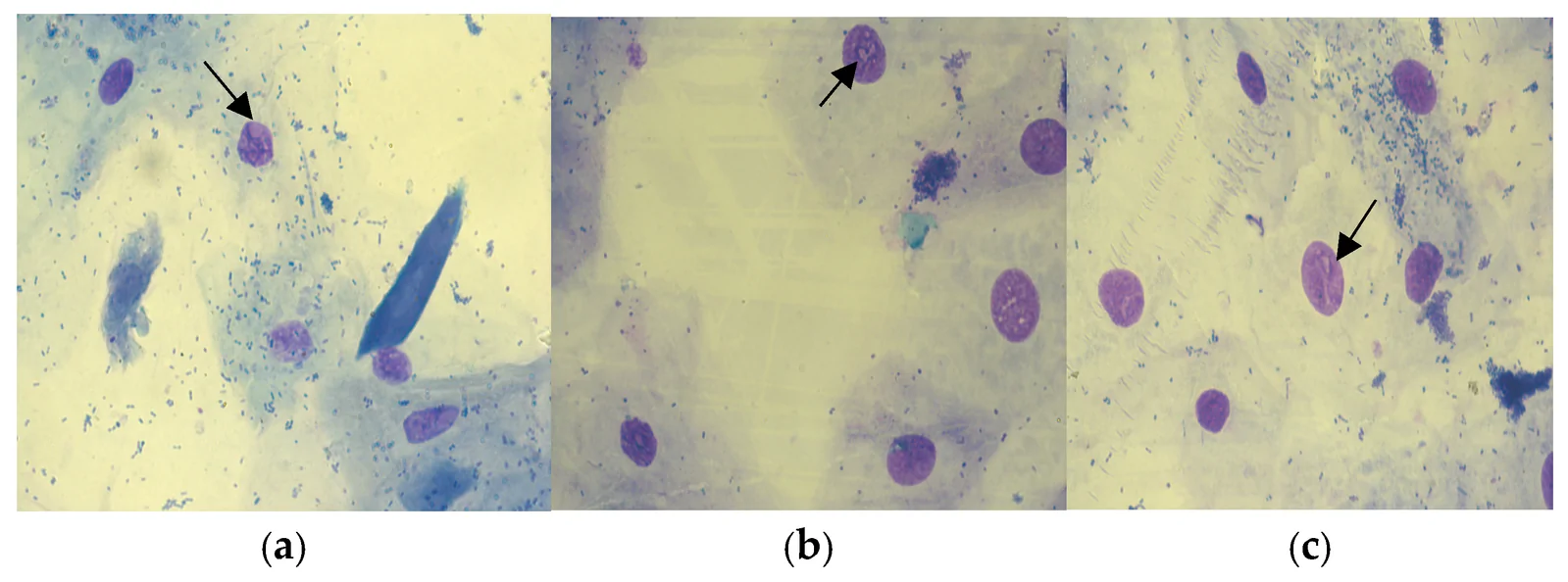
(**a**) Nuclear vacuolization in the buccal epithelial cytogram, Group I (T2DM). May–Grünwald eosin-methylene blue staining with Romanowsky azure-eosin counterstain. Magnification ×1000. (**b**) Nuclear vacuolization in the buccal epithelial cytogram, Group II (MS with impaired carbohydrate metabolism, prediabetes). May–Grünwald eosin-methylene blue staining with Romanowsky azure-eosin counterstain. Magnification ×1000. (**c**) Nuclear vacuolization in the buccal epithelial cytogram, Group III (MS without impaired carbohydrate metabolism, comparison group). May–Grünwald eosin-methylene blue staining with Romanowsky azure-eosin counterstain. Magnification ×1000.

The median frequency of nuclear vacuolization in the buccal epithelial cytogram of patients with T2DM was 1.5- and 2.5-fold higher than in patients with MS and prediabetes (*p* < 0.001) and in patients with MS without impaired carbohydrate metabolism (*p* < 0.001), respectively (Table 5, Figure 6a–c and Figure 13). The difference in the median frequency of nuclear vacuolization between patients with MS and prediabetes and patients with MS without impaired carbohydrate metabolism was also statistically significant (*p* < 0.001).

**Figure 7:**
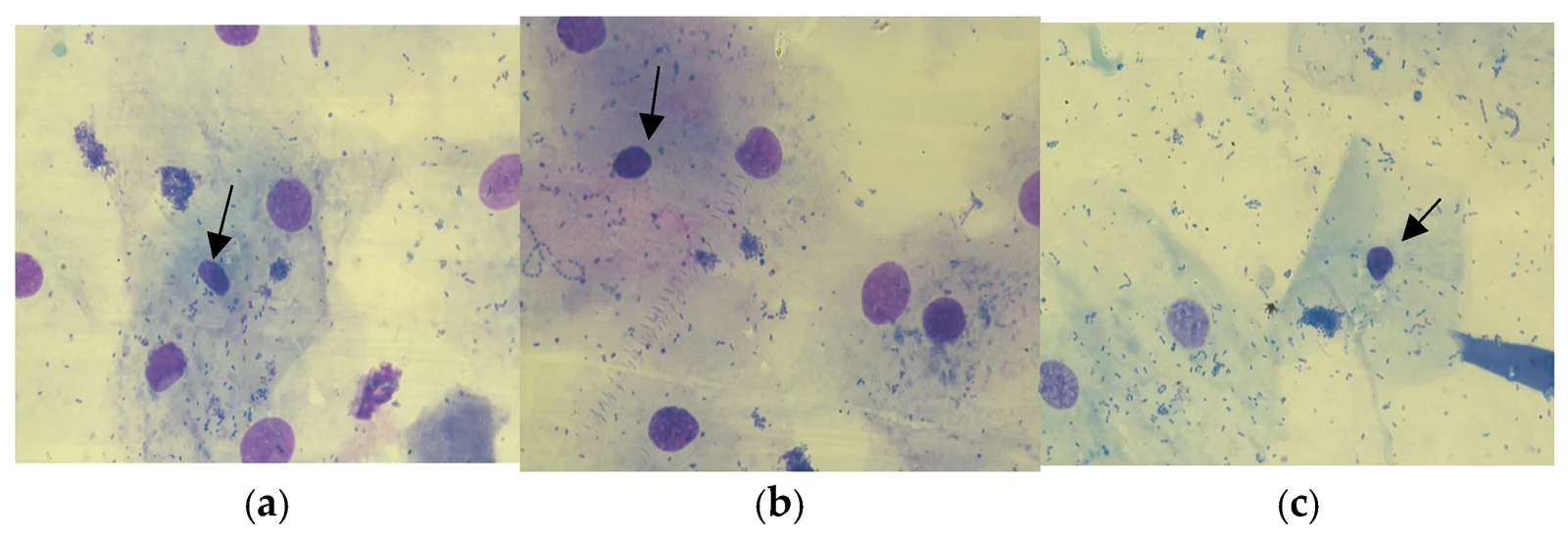
(**a**) Karyopyknosis in the buccal epithelial cytogram, Group I (T2DM). May–Grünwald eosin-methylene blue staining with Romanowsky azure-eosin counterstain. Magnification ×1000. (**b**) Karyopyknosis in the buccal epithelial cytogram, Group II (MS with impaired carbohydrate metabolism, prediabetes). May–Grünwald eosin-methylene blue staining with Romanowsky azure-eosin counterstain. Magnification ×1000. (**c**) Karyopyknosis in the buccal epithelial cytogram, Group III (MS without impaired carbohydrate metabolism, comparison group). May–Grünwald eosin-methylene blue staining with Romanowsky azure-eosin counterstain. Magnification ×1000.

The median frequency of karyopyknosis (shrunken, condensed nuclei) in the buccal epithelial cytogram was significantly lower in patients with T2DM than in patients with MS without impaired carbohydrate metabolism (*p* = 0.006) and did not differ significantly from that in patients with MS and prediabetes (*p* = 0.363) (Table 5, Figure 7a–c and Figure 10).

**Figure 8:**
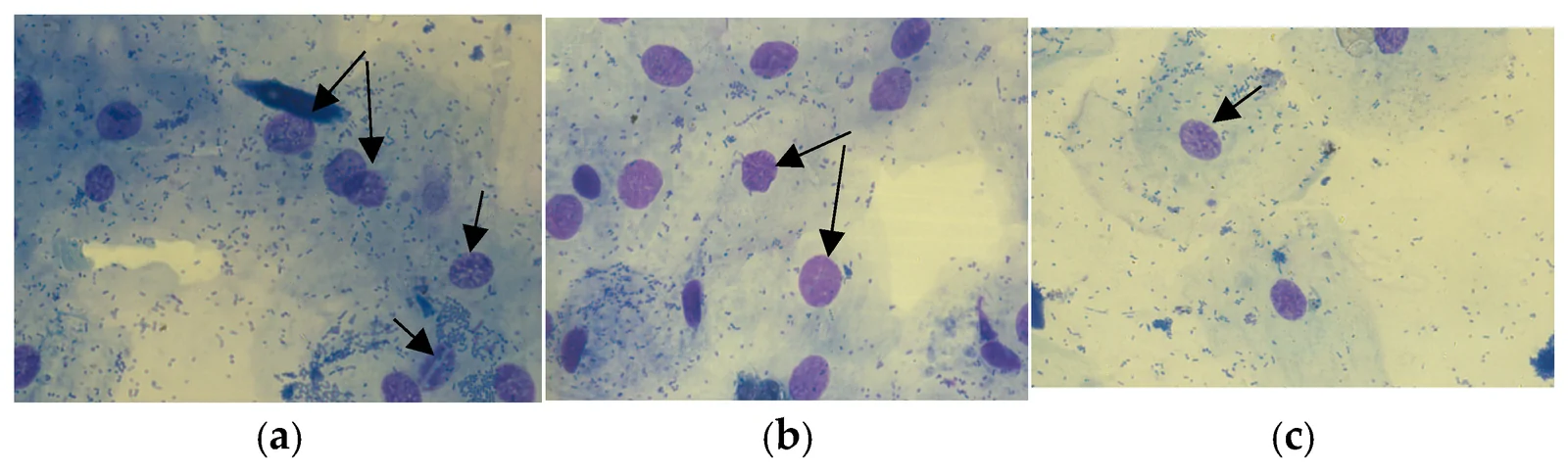
(**a**) Karyorrhexis in the buccal epithelial cytogram, Group I (T2DM). May–Grünwald eosin-methylene blue staining with Romanowsky azure-eosin counterstain. Magnification ×1000. (**b**) Karyorrhexis in the buccal epithelial cytogram, Group II (MS with impaired carbohydrate metabolism, prediabetes). May–Grünwald eosin-methylene blue staining with Romanowsky azure-eosin counterstain. Magnification ×1000. (**c**) Karyorrhexis in the buccal epithelial cytogram, Group III (MS without impaired carbohydrate metabolism, comparison group). May–Grünwald eosin-methylene blue staining with Romanowsky azure-eosin counterstain. Magnification ×1000.

The median frequency of karyorrhexis (nuclear fragmentation) in the buccal epithelial cytogram was 34-fold higher in patients with T2DM (*p* < 0.001) and 6-fold higher in patients with MS and prediabetes (*p* = 0.009) than in patients with MS without impaired carbohydrate metabolism (Table 5, Figure 8a–c and Figure 12). The difference between the T2DM and prediabetes groups was also statistically significant (*p* < 0.001).

**Figure 9:**
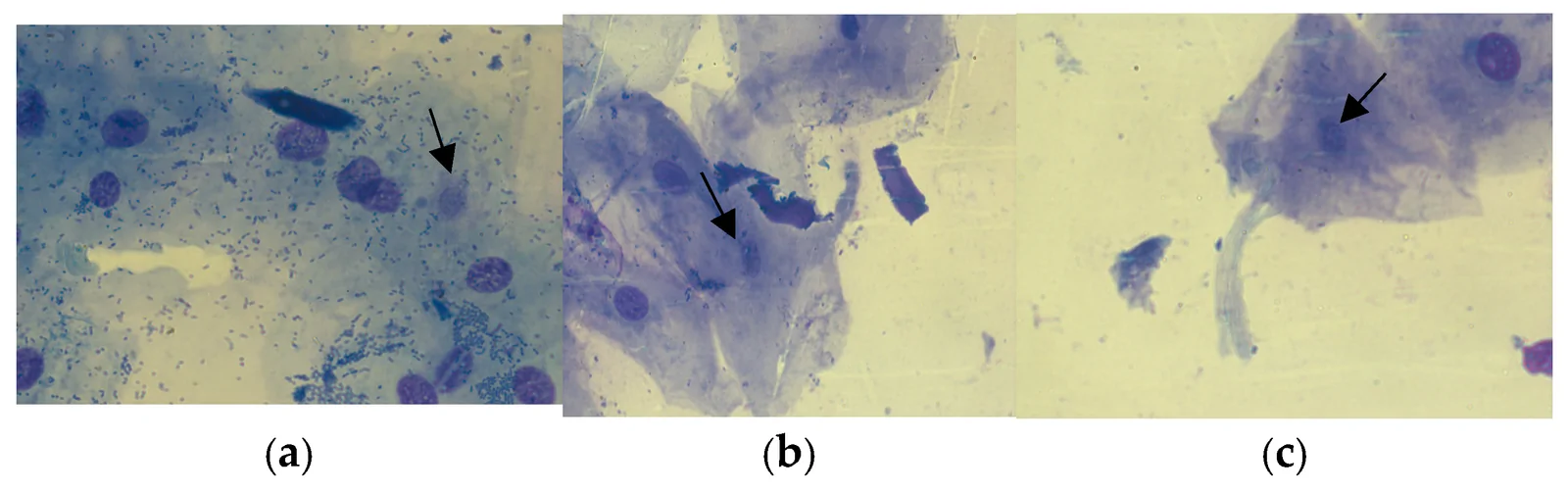
(**a**) Karyolysis in the buccal epithelial cytogram, Group I (T2DM). May–Grünwald eosin-methylene blue staining with Romanowsky azure-eosin counterstain. Magnification ×1000. (**b**) Karyolysis in the buccal epithelial cytogram, Group II (MS with impaired carbohydrate metabolism, prediabetes). May–Grünwald eosin-methylene blue staining with Romanowsky azure-eosin counterstain. Magnification ×1000. (**c**) Karyolysis in the buccal epithelial cytogram, Group III (MS without impaired carbohydrate metabolism, comparison group). May–Grünwald eosin-methylene blue staining with Romanowsky azure-eosin counterstain. Magnification ×1000.

**Figure 10:**
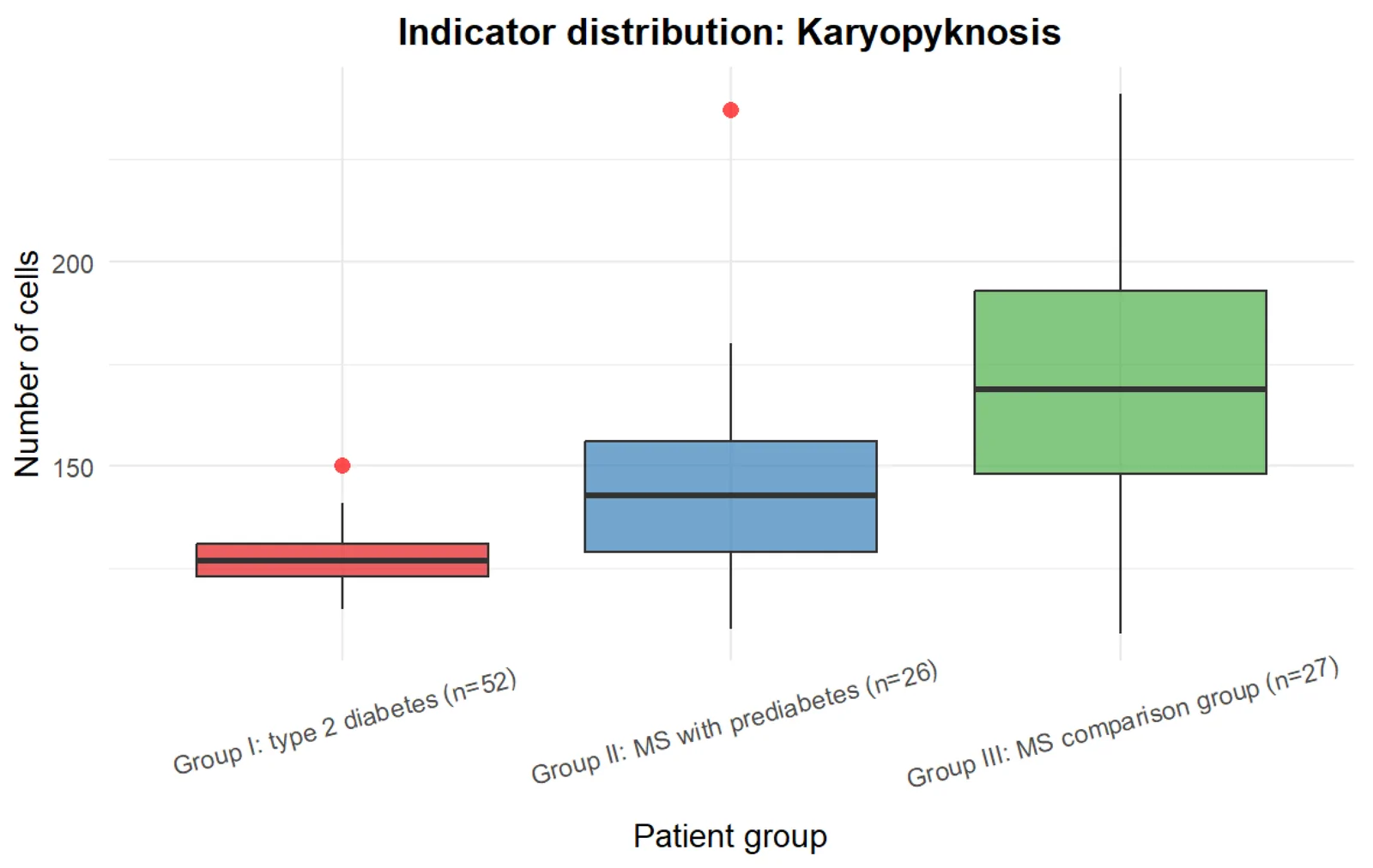
Distribution of the karyopyknosis frequency (number of cells per 1000 cells) in the buccal epithelial cytogram by study group (Group I—T2DM; Group II—MS with impaired carbohydrate metabolism, prediabetes; Group III—MS without impaired carbohydrate metabolism, comparison group).

**Figure 11:**
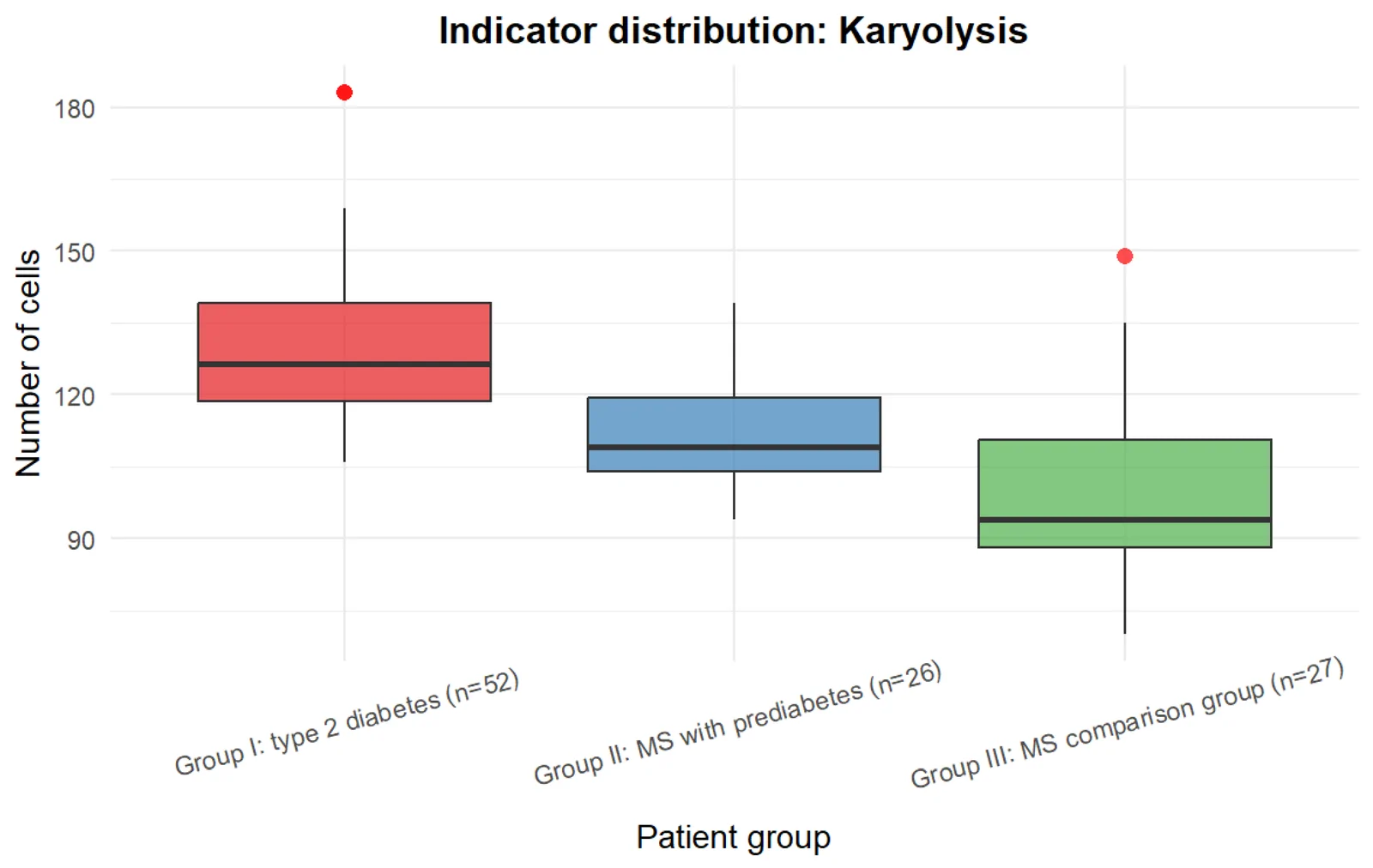
Distribution of the karyolysis frequency (number of cells per 1000 cells) in the buccal epithelial cytogram by study group (groups as in Figure 10).

**Figure 12:**
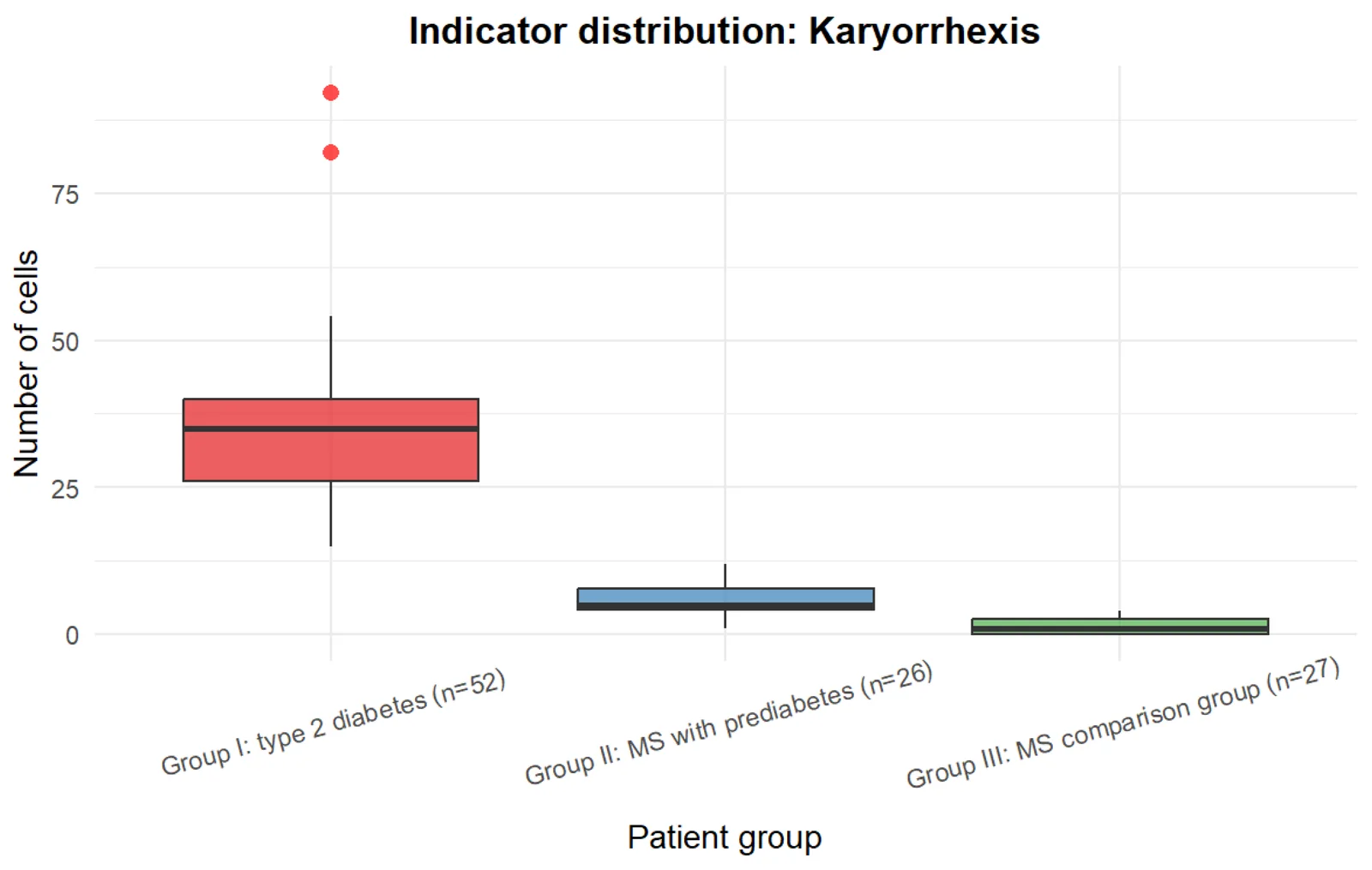
Distribution of the karyorrhexis frequency (number of cells per 1000 cells) in the buccal epithelial cytogram by study group (groups as in Figure 10).

**Figure 13:**
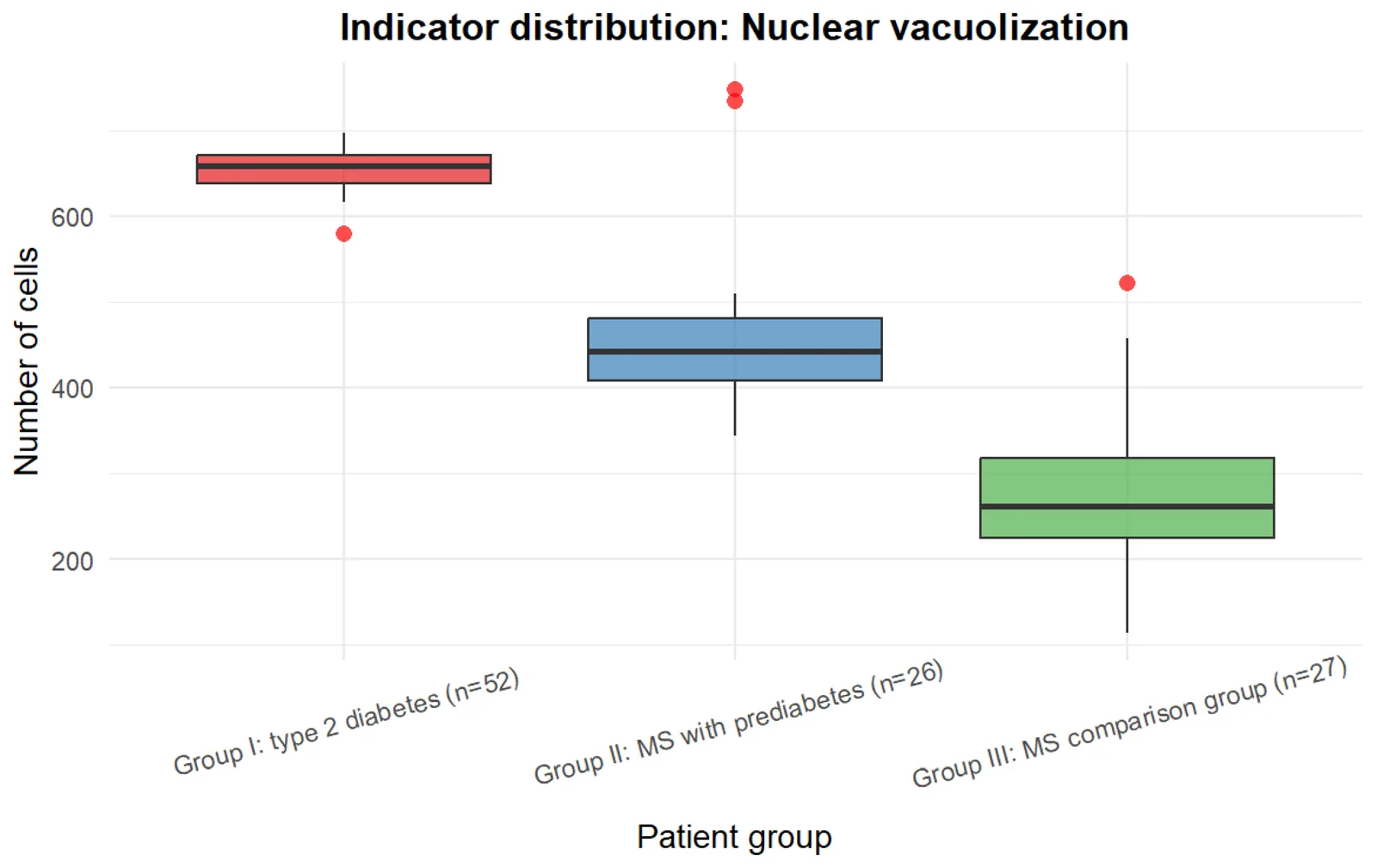
Distribution of the nuclear vacuolization frequency (number of cells per 1000 cells) in the buccal epithelial cytogram by study group (groups as in Figure 10).

**Figure 14:**
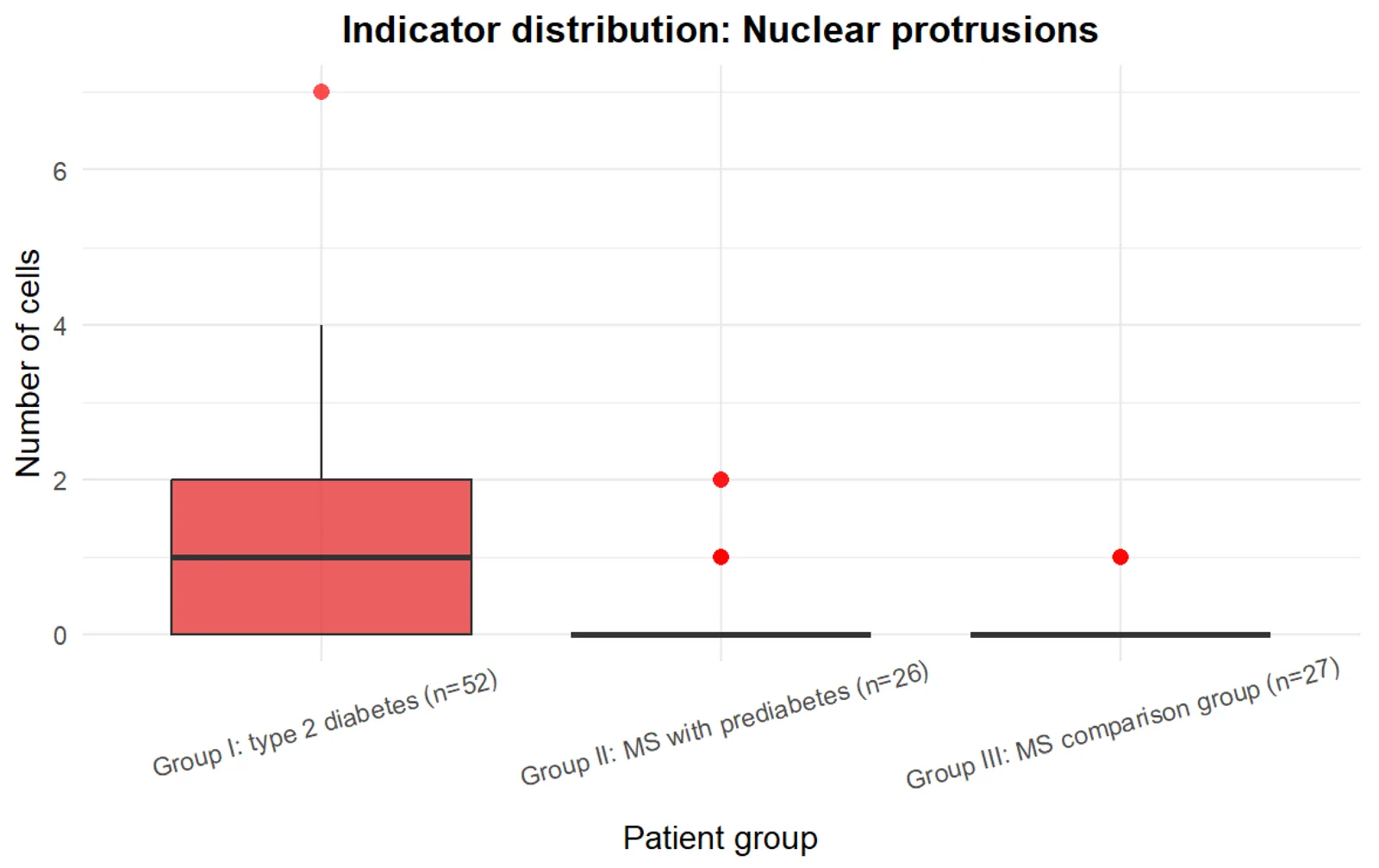
Distribution of the frequency of nuclear protrusions (micronucleus-like bodies, number of cells per 1000 cells) in the buccal epithelial cytogram by study group (groups as in Figure 10).

The median frequency of karyolysis in the buccal epithelial cytogram was significantly higher in patients with T2DM than in patients with MS without impaired carbohydrate metabolism (*p* = 0.014) and did not differ significantly from that in patients with MS and prediabetes (*p* = 0.142) (Table 5, Figure 9a–c and Figure 11).

**Table 5:** Median frequency (interquartile range) of apoptosis and cell necrosis/nuclear destruction in the buccal epithelial cytogram per 1000 cells.

Cytogram Indicators of BE	Group I, T2DM, *n* = 52	Group II, MS with Impaired Carbohydrate Metabolism (Prediabetes), *n* = 26	Group III, MS Without Impaired Carbohydrate Metabolism (Comparison Group), *n* = 27	Kruskal–Wallis Test	Dunn’s Post hoc Tests
Nuclear vacuolization	652 (632; 665)	446 (403; 488)	255 (212; 304)	*p* < 0.001	p_1,2_ < 0.001 * p_2,3_ < 0.001 * p_1,3_ < 0.001 *
Karyopyknosis	130 (122; 132)	144 (131; 158)	176 (162; 204)	*p* = 0.019	p_1,2_ = 0.363 p_2,3_ = 0.051 p_1,3_ = 0.006 *
Karyorrhexis	34 (25; 38)	6 (4; 8)	1 (0; 3)	*p* < 0.001	p_1,2_ < 0.001 * p_2,3_ = 0.009 * p_1,3_ < 0.001 *
Karyolysis	127 (116; 137)	108 (101; 118)	94 (88; 105)	*p* = 0.031	p_1,2_ = 0.142 p_2,3_ = 0.218 p_1,3_ = 0.014 *

Notes: BE—buccal epithelium; MS—metabolic syndrome; T2DM—type 2 diabetes mellitus. p_1,2_, p_2,3_ and p_1,3_—pairwise comparisons between Groups I and II, II and III, and I and III, respectively. *—differences are statistically significant after adjustment for multiple comparisons (*p* < 0.016).

Spearman’s rank correlation coefficients were also calculated between HbA1c levels and the indices of cell apoptosis and necrosis in the buccal epithelial cytogram, revealing significant correlations for nuclear vacuolization (ρ = 0.615, *p* < 0.001), karyopyknosis (ρ = −0.384, *p* = 0.005), karyorrhexis (ρ = 0.511, *p* < 0.001), and karyolysis (ρ = 0.269, *p* = 0.037). The negative correlation observed for karyopyknosis is consistent with its lower frequency in the groups with more severe glycemic impairment (Table 5), the inverse of the pattern seen for vacuolization, karyorrhexis, and karyolysis. Significant correlations were also found between glucose levels in unstimulated mixed oral fluid and the indices of cell apoptosis and necrosis in the buccal epithelial cytogram: nuclear vacuolization (ρ = 0.687, *p* < 0.001), karyopyknosis (ρ = −0.619, *p* < 0.001), karyorrhexis (ρ = 0.587, *p* < 0.001), and karyolysis (ρ = 0.499, *p* < 0.001). As with HbA1c, karyopyknosis was inversely correlated with oral fluid glucose, consistent with the lower karyopyknosis counts observed in patients with more advanced glycemic impairment.

In the exploratory compositional analysis, PERMANOVA indicated differences among the three groups in the relative distribution of normal cells, karyopyknosis, karyorrhexis, and karyolysis (counts per 1000 cells) (*p* < 0.001). Post hoc pairwise PERMANOVA tests with Bonferroni correction suggested differences between all pairs of groups (I vs. II, *p* = 0.008; II vs. III, *p* = 0.014; I vs. III, *p* = 0.0011). Given the methodological limitations of this analysis (a non-exhaustive sub-composition and the presence of zero counts without zero replacement; see Section Study Limitations), these results should be regarded as exploratory and descriptive only and were not used to support the conclusions of the study.

## 4. Discussion

Kumar H.N. et al. (2023) [11] reported binucleated cells in the buccal epithelial cytogram in 36.6% of individuals with type 2 diabetes mellitus, a frequency higher than in healthy individuals. This is indirectly consistent with the data of our study, in which binucleated cells were significantly more frequent in patients with metabolic syndrome and prediabetes than in patients of the comparison group (metabolic syndrome without impaired carbohydrate metabolism; *p* < 0.01).

Sahay K. et al. (2017) [12] observed cytoplasmic vacuolization in individuals with type 2 diabetes mellitus but not in healthy individuals. In our study, the median frequency of nuclear vacuolization in patients with T2DM was 1.5- and 2.5-fold higher than in patients with metabolic syndrome and prediabetes (*p* < 0.001) and in patients with metabolic syndrome without impaired carbohydrate metabolism (*p* < 0.001), respectively. It should be noted that Sahay K. et al. compared patients with healthy individuals, whereas in our study the comparison group consisted of individuals with metabolic syndrome without impaired carbohydrate metabolism, which may explain the differences between the findings.

According to Sahay K. et al. (2017) [12], karyorrhexis, karyolysis, and karyopyknosis were present only in individuals with diabetes mellitus and not in healthy individuals. This is partially consistent with the data of our study: karyorrhexis was 6-fold more frequent in patients with metabolic syndrome and prediabetes than in patients with metabolic syndrome without impaired carbohydrate metabolism (*p* < 0.01), and karyolysis was more frequent in patients with type 2 diabetes mellitus than in the comparison group (*p* = 0.014) but did not differ from that in patients with metabolic syndrome and prediabetes (*p* > 0.1).

In the study by Seifi S. et al. (2014) [2], binucleated cells, karyorrhexis, and nuclear vacuolization were significantly more frequent in individuals with diabetes mellitus than in healthy individuals, whereas no significant differences in cytological characteristics were found between patients with type 1 and type 2 diabetes. Similarly, Kumar H.N. et al. (2023) [11] reported that binucleated cells were significantly more frequent in oral epithelial smears from individuals with type 2 diabetes mellitus than from healthy individuals.

Diabetes mellitus has been reported to be an independent factor associated with changes in the cells of the oral mucosa [13]. In the present study, type 2 diabetes mellitus and prediabetes in patients with metabolic syndrome were associated with a higher frequency of degenerative changes in buccal epithelial cells, which is consistent with the data obtained by Agrawal R. et al. (2018) [14]. However, this association should not be interpreted as evidence of a direct causal effect of hyperglycemia: the oral hygiene (OHI-S) and periodontal inflammation (PMA) indices were markedly worse in the groups with impaired carbohydrate metabolism, particularly in the T2DM group, and no multivariable adjustment for these factors was performed. Local inflammation and poor oral hygiene may therefore have contributed to, or partly explained, the observed between-group differences in the cytograms, and an independent contribution of hyperglycemia remains to be established.

Excess glucose in the blood and tissue fluids may trigger a cascade of biochemical, metabolic, and structural changes in the epithelial cells of the oral mucosa, which, according to Narukawa Y. (2023), can be conditionally attributed to direct metabolic effects (glucotoxicity), oxidative stress and DNA damage, protein glycation, paracrine effects, and immune-inflammatory effects [15]. Our study showed an association between patient HbA1c level and nuclear vacuolization (ρ = 0.615, *p* < 0.001), karyopyknosis (ρ = −0.384, *p* = 0.005), karyorrhexis (ρ = 0.511, *p* < 0.001), and karyolysis (ρ = 0.269, *p* = 0.037) of buccal epithelial nuclei, which is consistent with the study of Zhang P. (2021), who explained this by the conversion of glucose to sorbitol under the action of aldose reductase and the accumulation of sorbitol inside cells, increasing osmotic pressure, which subsequently leads to intracellular edema, vacuolization of the cytoplasm and accelerated cellular aging [16]. In contrast, karyopyknosis was inversely correlated with HbA1c (ρ = −0.384, *p* = 0.005) and with oral fluid glucose (ρ = −0.619, *p* < 0.001), consistent with its lower frequency in the more glycemically severe groups (Table 5); this inverse pattern may reflect a shift away from classical pyknotic nuclear condensation towards karyorrhexis and karyolysis as the predominant modes of nuclear degeneration under more severe hyperglycemia and warrants further mechanistic investigation. Such associations may also be explained by excessive glycosylation of intracellular proteins and disruption of their normal folding and of epithelial cell division [17]. We also found a positive correlation between the indices of cell apoptosis and necrosis in the buccal epithelial cytogram and the glucose level in unstimulated mixed oral fluid at the time of sampling. A possible explanation is that hyperglycemia markedly increases the glucose concentration in the oral fluid secreted by the salivary glands; the buccal epithelium is constantly bathed in this fluid, which may shift the balance towards nuclear destruction [18]. However, these correlations were calculated on the pooled study population and do not establish a causal relationship.

Chronically elevated blood and salivary glucose levels can lead to non-enzymatic glycation of membrane and structural epithelial proteins, forming advanced glycation end products (AGEs), which in turn can bind to their receptors (RAGE) on the surface of buccal cells [15]. This reduces the expression of intercellular adhesion molecules and, as a result, can disrupt tight junctions between cells; the epithelial barrier becomes permeable [18], and cells begin to exfoliate prematurely (accelerated desquamation) [19]. Mitochondrial overload with glucose causes excessive production of reactive oxygen species, and oxidative stress, in turn, damages the nuclear DNA of buccal cells [20]. However, the role of hyperglycemia-associated oxidative stress in the destruction of buccal epithelial cell nuclei was not specifically assessed in the present study.

### Future Directions

The directions outlined below do not follow directly from the present dataset; rather, they describe the studies that would be required to test the hypotheses generated here. As the present findings are preliminary and observational, several steps should be taken before buccal epithelial cytology can be considered as a screening indicator in clinical practice. First, longitudinal clinical follow-up of the study cohort, including patients whose metabolic syndrome without impaired carbohydrate metabolism progresses to prediabetes or type 2 diabetes, would clarify whether cytological abnormalities precede or track the onset of glycemic dysfunction. Second, prospective validation cohorts that include a healthy, non-MS control group are needed to calculate diagnostic accuracy metrics (ROC curves, sensitivity, specificity, and optimal cut-off values) for each cytological parameter and to adjust for oral hygiene and periodontal inflammation in multivariable models. Third, integrating buccal cytogram indices with salivary inflammation panels (e.g., IL-6, TNF-α, and C-reactive protein) may improve discrimination between metabolic syndrome subtypes and provide mechanistic insight into the interplay between hyperglycemia-driven inflammation and epithelial damage [21]. Finally, automated, AI-based cytometric image analysis could standardize the identification and quantification of nuclear aberrations, by reducing interobserver variability and enabling scalable, reproducible screening [22].

## 5. Conclusions

In this observational cross-sectional study, prediabetes and type 2 diabetes mellitus in patients with metabolic syndrome (MS) were associated with a higher frequency of cell damage at the division stage and nuclear abnormalities in the buccal epithelium than MS without impaired carbohydrate metabolism. These associations should be regarded as hypothesis-generating: because the oral hygiene and periodontal inflammation indices were also worse in the groups with impaired carbohydrate metabolism and no multivariable adjustment was performed, an independent effect of hyperglycemia cannot be inferred from the present data. The findings are preliminary and require further research to establish reference values for the parameters studied and to determine whether they can be used in the laboratory monitoring of patients with prediabetes and diabetes. Given the absence of a healthy control group and of formal diagnostic accuracy analysis, buccal epithelial cytology cannot currently be recommended as a screening or standalone diagnostic tool; it should be regarded only as a candidate non-invasive complementary indicator of impaired carbohydrate metabolism, whose clinical utility must be confirmed in prospective, controlled validation studies with predefined thresholds and estimates of diagnostic accuracy. The results obtained are preliminary, as the study we conducted should be interpreted strictly as exploratory and hypothesis-generating.

## 6. Study Limitations

This study was limited to patients with metabolic syndrome, which allowed us to evaluate the frequency of cell damage at the division stage, apoptosis, and cell necrosis/nuclear destruction in the buccal epithelial cytogram in relation to the presence and severity of impaired carbohydrate metabolism within a metabolically comparable population; however, a control group of healthy individuals (without MS) was not included in the study. In addition, group allocation was based on the retrospective stratification of medical records, whereas the cytological, oral fluid, and blood samples were collected prospectively at a single time point. Therefore, the temporal relationship between the onset of glycemic dysfunction and the cytological changes cannot be established, and selection bias related to the use of existing medical records cannot be excluded.

No a priori sample-size or statistical power calculation was performed; the sample size was determined by the clinical availability of eligible patients during the recruitment period, and the study should be regarded as exploratory rather than confirmatory. Furthermore, although pairwise post hoc comparisons within each cytological parameter were corrected for multiplicity using Dunn’s test, no correction was applied across the multiple cytological and biochemical endpoints examined in the study as a whole; some of the reported associations may therefore reflect the number of comparisons performed and should be interpreted cautiously pending confirmation in adequately powered, pre-registered studies. Correlation coefficients between glycated hemoglobin/glucose and cytological indices were computed on the pooled study population; because these correlations were not additionally verified within each clinical group separately, part of the observed association may reflect between-group separation (i.e., patients with T2DM having both higher glycemic values and higher rates of nuclear damage) rather than a continuous within-group relationship, and this should be taken into account when interpreting the strength of these associations. Data on diabetes duration, glucose-lowering treatment, and other systemic conditions or medications that could independently affect oral mucosal cytology were not systematically collected and could not be adjusted for in this analysis.

Interobserver variability in cytogram analysis was not planned for the study, so agreement measures (e.g., Cohen’s kappa) were not assessed. Cytology of buccal epithelial preparations for each patient was performed in parallel by two cytologists from a single medical center. In cases of disagreement regarding nuclear abnormalities, the slides were reviewed jointly by the two cytologists; no third expert was involved. No external validation of the preparations by cytologists from another medical center was performed. Additionally, the PMA and OHI-S indices of local oral inflammation/hygiene were markedly worse in the T2DM group compared with the comparison group; a multivariable regression analysis adjusting for these confounders was not performed in this exploratory study, so an independent contribution of hyperglycemia beyond the severity of local oral inflammation cannot be established from the present data, and the observed cytological abnormalities should be interpreted as associated with, rather than caused by, impaired carbohydrate metabolism. This should be addressed in future confirmatory studies.

This study focused on the direct metabolic and paracrine effects of hyperglycemia on changes in buccal epithelial cytograms. The roles of oxidative stress, DNA damage, and protein glycation were not assessed, as the study was preliminary (exploratory). The study also did not evaluate the role of proinflammatory cytokines (IL-6, IL-1β, and TNF-α) and the intracellular inflammasome (Caspase-1) in the destruction of buccal epithelial cell nuclei.

It should also be noted that the groups were imbalanced with respect to sex and, among men, age. The conclusions were therefore formulated cautiously, taking into account the heterogeneity of the groups. Because metabolic syndrome without impaired carbohydrate metabolism may progress to prediabetes and subsequently to type 2 diabetes, and because the risk of age-related diseases increases over time, some of the age differences between the groups may reflect the natural history of these conditions.

## Figures and Tables

**Table 1:** Demographic and clinical characteristics of the patients with metabolic syndrome components in the study groups.

Characteristics	Group I: Type 2 Diabetes Mellitus (*n* = 52)	Group II: MS with Impaired Carbohydrate Metabolism (Prediabetes) (*n* = 26)	Group III (Comparison Group): MS Without Impaired Carbohydrate Metabolism (*n* = 27)	*p*-Level
Gender (abs/%)	F: 25 (48.1%) M: 27 (51.9%)	F: 20 (76.9%) M: 6 (23.1%)	F: 24 (88.9%) M: 3 (11.1%)	χ^2^ = 15.065. <0.001 ***
Age (years): by gender (F/M)	36.5 (25.5–53)	38 (36–52)	38.5 (35–50)	0.061
	39 (26–54) 34 (25–53)	40.5 (36–54) 35.5 (29–48)	41 (35–54) 36 (27–43)	0.059 0.048 *
BMI: by gender (F/M)	35 (25.8–42.5)	32.1 (30.8–39.5)	31.1 (30.5–39.1)	0.112
	34.1 (25.8–42.4) 35.9 (25.6–42.6)	32.8 (32.8–41.2) 31.3 (26.2–29.4)	31.5 (30.5–40.2) 30.7 (25.5–33.8)	0.198 0.075
Waist circumference (cm): by gender (F/M)	111 (105–124)	106 (98–116)	104 (96–112)	0.073
	105 (98–119) 117.4 (111–128)	105.4 (95–115) 112.8 (107–121)	101.5 (94–110) 109 (105–114)	0.059 0.095
Fasting venous plasma glucose (mmol/L) by gender (F/M)	10.6 (7.0–14.2)	5.2 (4.6–5.5)	4.65 (4.3–5.3)	<0.001 ***
	10.8 (6.6–14.4) 10.4 (7.2–14)	5.2 (4.5–5.5) 5.2 (4.8–5.4)	4.8 (4.3–5.4) 4.5 (3.5–4.5)	<0.001 *** <0.001 ***
2 h post-load venous plasma glucose (mmol/L) by gender (F/M)	9.1 (8.0–9.8)	8.1 (7.9–8.7)	6.2 (5.4–6.9)	<0.001 ***
	9.14 (8.0–10) 9.0 (8.0–9.5)	8.2 (7.9–8.8) 7.9 (7.9–8.2)	6.3 (5.4–6.9) 6.1 (5.6–6.7)	<0.001 *** 0.003 **
Glycated hemoglobin (HbA1c), %	9.7 (8.8; 10.5)	6.3 (5.2; 7.3)	5.5 (5.1; 6.1)	0.001 **
High-sensitivity C-reactive protein	8.0 (5.5; 11.5)	5.0 (2.0; 9.8)	3.8 (1.6; 6.5)	<0.001 ***
Smoking	6 (11%)	3 (11%)	4 (15%)	0.906

*, **, ***—differences are significant at *p* < 0.05, *p* < 0.01, and *p* < 0.001, respectively.

**Table 2:** Oral hygiene and periodontal indices in the study groups (Kruskal–Wallis test).

Index	Group I: Type 2 Diabetes Mellitus (*n* = 52)	Group II: MS with Impaired Carbohydrate Metabolism (Prediabetes) (*n* = 26)	Group III (Comparison group): MS Without Impaired Carbohydrate Metabolism (*n* = 27)	*p*-Level (Kruskal–Wallis Test)
OHI-S index	3.5 (3.0–4.0)	2.5 (2.0–3.0)	2 (1.75–2.0)	0.009 **
PMA index (Parma, 1960)	61.0 (60.0–65.0)	48.75 (48–49.75)	9.75 (9.5–10.5)	<0.001 ***

**, ***—differences are significant at *p* < 0.01 and *p* < 0.001, respectively.

**Table 3:** Glucose levels in unstimulated mixed oral fluid in the study groups.

Indicator/Group	Group I: Type 2 Diabetes Mellitus (*n* = 52)	Group II: MS with Impaired Carbohydrate Metabolism (Prediabetes) (*n* = 26)	Group III (Comparison Group): MS without Impaired Carbohydrate Metabolism (*n* = 27)	*p*-Level (Kruskal–Wallis U Test)
Glucose in unstimulated mixed oral fluid, µmol/L	800 (500; 1600)	300 (200; 550)	150 (100; 250)	p_1,2_ < 0.001 ** p_2,3_ = 0.014 * p_1,3_ < 0.001 **

*, **—differences are statistically significant after Bonferroni correction at *p* < 0.016 and *p* < 0.001, respectively. p_1,2_—Group I vs. Group II; p_2,3_—Group II vs. Group III (comparison group); p_1,3_—Group I vs. Group III (comparison group).

**Table 4:** Median frequency (interquartile range) of cell damage at the division stage in the buccal epithelial cytogram per 1000 cells.

Cytogram Indicators of BE	Group I, T2DM, *n* = 52	Group II, MS with Impaired Carbohydrate Metabolism (Prediabetes), *n* = 26	Group III, MS Without Impaired Carbohydrate Metabolism (Comparison Group), *n* = 27	Kruskal–Wallis Test	Dunn’s Post hoc Tests
Normal cells	47 (42; 72)	239 (201; 284)	454 (396; 514)	*p* < 0.001	p_1,2_ < 0.001 * p_2,3_ < 0.001 * p_1,3_ < 0.001 *
Binucleated cells	5 (3; 9)	7 (4; 10)	1 (0; 2)	*p* = 0.042	p_1,2_ = 0.415 p_2,3_ = 0.008 * p_1,3_ = 0.062
Nuclear notches	29 (22; 35)	26 (21; 31)	15 (12; 24)	*p* = 0.024	p_1,2_ = 0.612 p_2,3_ = 0.048 p_1,3_ = 0.025
Nuclear protrusions (“nuclear buds”, micronucleus-like bodies)	1 (0; 2)	0 (0; 0)	0 (0; 0)	*p* = 0.021	p_1,2_ = 0.038 p_2,3_ = 0.411 p_1,3_ = 0.015 *

Notes: BE—buccal epithelium; MS—metabolic syndrome; T2DM—type 2 diabetes mellitus. p_1,2_, p_2,3_ and p_1,3_—pairwise comparisons between Groups I and II, II and III, and I and III, respectively. *—differences are statistically significant after adjustment for multiple comparisons (*p* < 0.016).

## Data Availability

The data presented in this study are available on request from the corresponding author because of ethical and privacy restrictions related to patient data.
